# Limitations of Baseline Impedance, Impedance Drop and Current for Radiofrequency Catheter Ablation Monitoring: Insights from In silico Modeling

**DOI:** 10.3390/jcdd9100336

**Published:** 2022-10-03

**Authors:** Ramiro M. Irastorza, Timothy Maher, Michael Barkagan, Rokas Liubasuskas, Juan J. Pérez, Enrique Berjano, Andre d’Avila

**Affiliations:** 1 Instituto de Física de Líquidos y Sistemas Biológicos (CONICET), La Plata B1904, Argentina; 2 Departamento de Ingeniería Mecánica, Facultad Regional La Plata, Universidad Tecnológica Nacional, La Plata C1041, Argentina; 3 Division of Cardiovascular Medicine, Harvard-Thorndike Electrophysiology Institute, Beth Israel Deaconess Medical Center, Harvard Medical School, Boston, MA 02115, USA; 4 Shamir Medical Center, Cardiology Division, Sackler School of Medicine, Tel Aviv University, Beer-Yakov 69978, Israel; 5 Department of Radiology, Beth Israel Deaconess Medical Center, Harvard Medical School, Boston, MA 02115, USA; 6 BioMIT, Department of Electronic Engineering, Universitat Politècnica de València, 46022 Valencia, Spain

**Keywords:** biophysics, computer modeling, impedance, radiofrequency catheter ablation

## Abstract

Background: Baseline impedance, radiofrequency current, and impedance drop during radiofrequency catheter ablation are thought to predict effective lesion formation. However, quantifying the contributions of local versus remote impedances provides insights into the limitations of indices using those parameters. Methods: An in silico model of left atrial radiofrequency catheter ablation was used based on human thoracic measurements and solved for (1) initial impedance (*Z*), (2) percentage of radiofrequency power delivered to the myocardium and blood (3) total radiofrequency current, (4) impedance drop during heating, and (5) lesion size after a 25 W–30 s ablation. Remote impedance was modeled by varying the mixing ratio between skeletal muscle and fat. Local impedance was modeled by varying insertion depth of the electrode (ID). Results: Increasing the remote impedance led to increased baseline impedance, lower system current delivery, and reduced lesion size. For ID = 0.5 mm, *Z* ranged from 115 to 132 Ω when fat percentage varied from 20 to 80%, resulting in a decrease in the RF current from 472 to 347 mA and a slight decrease in lesion size from 5.6 to 5.1 mm in depth, and from 9.2 to 8.0 mm in maximum width. In contrast, increasing the local impedance led to lower system current but larger lesions. For a 50% fat–muscle mixture, *Z* ranged from 118 to 138 Ω when ID varied from 0.3 to 1.9 mm, resulting in a decrease in the RF current from 463 to 443 mA and an increase in lesion size, from 5.2 up to 7.5 mm in depth, and from 8.4 up to 11.6 mm in maximum width. In cases of nearly identical *Z* but different contributions of local and remote impedance, markedly different lesions sizes were observed despite only small differences in RF current. Impedance drop better predicted lesion size (R^2^ > 0.93) than RF current (R^2^ < 0.1). Conclusions: Identical baseline impedances and observed RF currents can lead to markedly different lesion sizes with different relative contributions of local and remote impedances to the electrical circuit. These results provide mechanistic insights into the advantage of measuring local impedance and identifies potential limitations of indices incorporating baseline impedance or current to predict lesion quality.

## 1. Introduction

**Figure 1:**
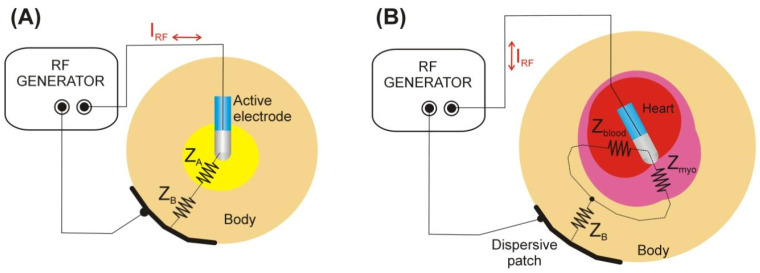
(**A**) Lumped elements electrical model demonstrating that total impedance in RFCA can be modeled as two impedances in series with the tissue around the active electrode *Z_A_* (myocardium and circulating blood) and that associated with the rest of the ‘remote’ tissues *Z_B_* (including those near the dispersive patch). (**B**) *Z_A_* is comprised of two parallel impedances, one associated with the blood (*Z*_*blood*_) and the other with the myocardium (*Z*_*myo*_).

Radiofrequency catheter ablation (RFCA) to treat arrhythmias depends on forming large enough lesions to eliminate the arrhythmogenic substrate or create durable lines of conduction block. It has been previously reported that baseline impedance (*Z*) impacts lesion size [1,2,3] and tissue temperature [4] during radiofrequency catheter ablation (RFCA) with constant power (*P*). A lower impedance leads to increased output current (*I*) due to the inverse relationship *P = I*^2^ × *Z*. The more current that is distributed at the ablation site, the more heating that is produced and the larger the lesion that is formed. The electrical circuit in RFCA is comprised of all the equipment and tissues through which RF current flows. As shown in Figure 1A, electrical impedance can be modeled as two impedances in series [5]: *Z_A_* is associated with the tissue around the active electrode (myocardium and circulating blood), while *Z_B_* is associated with the rest of the tissues (including those near the dispersive patch) [6]. The overall *Z* is calculated as *Z_A_* + *Z_B_*. Moreover, *Z_A_* is comprised of two parallel impedances, one associated with the blood (*Z_blood_*) and other with the myocardium (Z*_myo_*) [6] (Figure 1B). Only the current dissipated at Z*_myo_* contributes to the lesion size, while current dissipated through blood does not, implying ‘lost’ RF energy. Since electrical conductivity of myocardium is lower than blood [7], *Z_A_* increases as the electrode is inserted further into the myocardium as greater contact force is applied. An ablation electrode floating freely in the cardiac chamber (with contact force of 0 g) would therefore have the lowest possible *Z* in a lumped element electrical model [5]. A lumped element electrical model is an important simplification of the real situation in which the domain is continuous and therefore the electrical variables are distributed throughout the tissues. To date, neither *Z_A_* nor *Z_B_* have been rigorously quantified in spatial terms. Instead, it has been assumed that they correspond to near and far impedance, respectively, relative to the ablation site. Their differences have been used to explain variations in heating and lesion size.

Previous experimental studies concluded that lower impedance leads to increased tissue heating and larger lesion sizes [1,2] due to increased current delivery. During these experiments, baseline impedance was adjusted by exclusively modifying the remote component of the impedance, i.e., varying *Z_B_*. This was performed in different ways, such as using serial resistors [1], choosing different locations for the patches [2], or by altering the extent of contact of the return electrode [2]. That study did not assess the effect of varying the baseline impedance due to changes near the ablation site, i.e., variations in *Z_A_*. For example, an RF electrode inserted deep into a crevice could lead to a high value of *Z_A_*, contributing to a high value of the baseline impedance but still allowing for a large RF lesion. We hypothesize that the baseline impedance does not always predict the amount of power dissipated around the RF electrode, and therefore does not necessarily predict the resulting lesion size. Depending on the relative contributions of *Z_A_* and *Z_B_* to the baseline impedance, the electrical behavior will differ significantly.

Since baseline impedance can affect power delivery to tissues, some have advocated for titrating power based on the baseline impedance to create otherwise similar lesion sizes. The concept of ‘corrected power’ has been suggested in [2], and consists of programming the power (*P*) according with the value of the baseline impedance *Z* as follows: *P(corr)* = 40 W × (*Z*/120 Ω) in order to obtain the same lesion size as would be obtained if the 40 W were delivered with a baseline impedance of 120 Ω (which is a typical baseline impedance seen in clinical practice with RFCA in the left atrium). The motivation is to prevent overheating when *Z* is much less than 120 Ω or provide the extra power needed when *Z* is much greater than 120 Ω. We hypothesize that this concept will not be effective under all circumstances and will only be helpful when *Z* differs from 120 Ω due to variations in the remote component *Z_B_*.

Controlling and precisely varying *Z_A_* and *Z_B_* in an in vivo experimental model would be technically challenging. Computer modeling, on the other hand, allows for controlling numerous variables to form predictions about complex biophysical systems, including RFCA [8,9]. We planned a study based on an in silico model to investigate the hypothesis that baseline impedance can independently predict lesion size in RFCA.

## 2. Methods

### 2.1. Model Description

In silico models are based on a geometry which is a simplification of the real physical scenario. The mathematical equations that represent the physical phenomena involved are computationally solved. In the case of RFCA, Laplace’s equation is used to compute the RF power deposited in the tissues while Bioheat equation allows solving the thermal conduction problem [8]. Using this methodology, we built 8500-element models and computed the: (1) Initial impedance, (2) Percentages of RF power delivered to the myocardium and blood, (3) Total RF current, (4) Impedance drop, and (5) Lesion size (using the 50 °C isoline) after a 25 W–30 s ablation.

**Figure 2:**
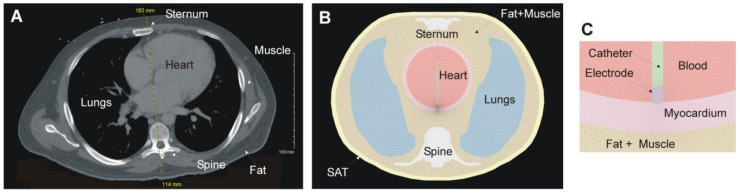
(**A**) CT-scan slice from a representative patient. (**B**) Computer model (units in mm) of posterior RFCA inspired by the CT-scan image and including the most representative organs. (**C**) Zoom of the RF catheter and cardiac wall.

The model anatomic schema was derived from a CT-scan slice of a representative patient (Figure 2A) and focused on simulating RFCA of the posterior left atrium (LA). The model included bony structures (spine and sternum) and lungs, all surrounded by a mix of muscle and fat (Figure 2B). The heart was modeled as a sphere (10 cm inner diameter) full of blood with a 4 mm shell mimicking the cardiac wall. Inside the cardiac chamber, an RF catheter was placed in perpendicular orientation on the posterior cardiac wall. The RF catheter comprised of a round-tip metal electrode (7 Fr, 3.5 mm) and a fragment of plastic tubing (catheter). The electrode irrigation was modeled by fixing a value of 45 °C in the cylindrical zone of the electrode tip, and leaving the semispherical tip free, mimicking a multi-hole electrode (assuming that irrigation occupies almost the entire surface of the electrode) [8]. The thermal problem was not feasible to solve in the blood pool, so thermal transfer coefficients for low blood flow condition (0.1 m/s) were used on the electrode-blood and myocardium-blood interfaces (3446 W/m^2^·K and 610 W/m^2^·K, respectively). Initial temperature was set at 37 °C. An electrical boundary condition of 0 V was set on the torso surface corresponding with the dispersive patch location, while the RF current through the electrode was modulated during the ablation to keep power constant. The model solved a coupled electric-thermal problem numerically using the Fenics software [10] and Gmsh mesher [11]. Details of these equations and boundary conditions are described in detail elsewhere [8].

**Figure 3:**
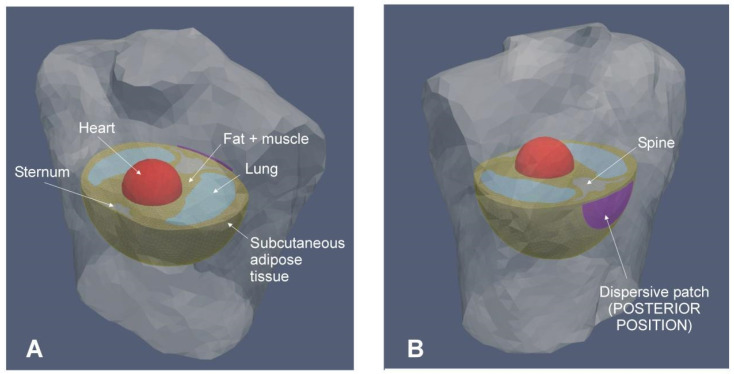
Anterior (**A**) and posterior (**B**) views of the patient’s torso including the elements included in the computational model.

To allow for the calculations to be performed, the created model was two dimensional with axial symmetry, with the volumes corresponding to the organs created by rotation around the axis of the RF catheter, as illustrated in Figure 3. To maintain the axial symmetry, the dispersive patch was assumed to be a 7 cm radius disk placed on the posterior side, with a contact area of 154 cm^2^ (which is a value very similar to the commercially available dispersive patch). The heart was placed in such a way that the distances between the electrode and the posterior and anterior sides were exactly 117 and 154 cm, respectively, which corresponds with the mean values measured in a retrospective sample of 20 patients undergoing a CT scan of the torso [12]. This study conformed to the guidelines of the local institutional review board. The electrical and thermal properties of the tissues were taken from the IT’IS Foundation database [7] while the ablation catheter properties were taken from [13]. Table 1 shows the characteristics of the materials used in the model. The values for lung were the mean between inflated and deflated. The values for bone (spine and sternum) were the mean between cortical and trabecular bone. The values for the tissue surrounding organs were the mean between muscle and subcutaneous fat. The 6 mm outer layer was assumed to be subcutaneous fat.

### 2.2. Modeling the Dispersion of ‘Remote’ Impedance Z_B_

The mixing ratio between skeletal muscle and fat in the tissues surrounding the organs were varied from 20 to 80% in order to simulate the variability found in patients. Five cases were considered: 20%, 35%, 50%, 65% and 80% fat percentage. This ranged was derived from the analysis of the CT-scan images reported in [12], which were segmented to identify the percentage of fat and muscle around heart, lungs and bony structures using *Scikit-image*, which comprised of a collection of image processing algorithms implemented in the Python programming language [14]. The mixing ratio between skeletal muscle and fat in the tissues surrounding the organs affects only the remote part of the baseline impedance, *Z_B_*, since these tissues are far from the lesion site. Due to the lower electrical conductivity of fat compared to muscle (0.0438 vs. 0.446 S/m) [7], *Z_B_* will be larger as the percentage of fat increases.

### 2.3. Modeling the Dispersion of ‘Local’ Impedance Z_A_

The insertion depths of the electrode into the tissue varied from 0.3 to 1.9 mm, which mimics a broad range of contact surfaces between tissue and electrode (Figure 2C). While a range of 0.3–0.7 mm would mimic low contact forces (5–20 g) [9], values as high as 1.9 mm would mimic a large electrode-coverage level as described by Bourier et al. [15], i.e., with the electrode deeply inserted into the tissue. As the insertion depth of the RF electrode increased from 0.3 to 1.9 mm, the *Z_A_* value increased accordingly since the electrical conductivity of the myocardium is lower than that of blood (0.281 vs. 0.748 S/m) [7]. Note that changes in catheter orientation (perpendicular, parallel, and oblique) could also affect the contact surface and hence the *Z_A_* value. The simulations in this study allowed both *Z_A_* and *Z_B_* to be varied at the same time, achieving in some cases identical baseline impedance values (*Z_A_* + *Z_B_*), allowing a critical assessment of the concept that a specific value of baseline impedance determines the size lesion.

### 2.4. The Concept of ‘Corrected Power’

To assess the ‘corrected power’ concept [2], we compared the lesion sizes computed for different baseline impedance (*Z*) values under two conditions: (1) ‘non-corrected power’, using 25 W, and (2) ‘corrected power’, i.e., using a *Z*-dependent power according with 25 W × (*Z*/120 Ω). The different values of *Z* were achieved in two ways: (1) varying the insertion depth (from 0.3 to 1.3 mm) for the same patient (50% fat, one patch), which implies changing *Z_A_* while *Z_B_* remains constant, and (2) changing the % of fat for the same insertion depth of 0.5 mm, which implies changing *Z_B_* while *Z_A_* remains constant.

### 2.5. Statistics

This study used a physics-based mechanistic model. We assumed an uncertainty in the ratio between skeletal muscle and fat in the tissues surrounding the organs, and in the insertion depths of the electrode into the tissue, as detailed above. This provided 45 unique cases that could represent a representative sample of what happens during ablation of the RFCA under varying conditions. The relationships between the variables were studied by simple regression using Excel. Coefficient of determination (R^2^) was reported to assess the goodness of fit, along with the *p*-value (*p*) to determine the statistically significance.

## 3. Results

### 3.1. Morphological Data from the CT Scans

The mixing ratio between skeletal muscle and fat in the tissues surrounding the organs was estimated from the analysis of the CT-scan images reported in [12]. Table 2 shows the morphological data of the analyzed patients, along with the mixing ratio of fat relative to skeletal muscle. This analysis provided a 29% minimum, 81% maximum, 61% mean and 14% standard deviation. The range of mixing ratio chosen for the simulations (20–80%) was used in order to generate applicable results to clinical practice.

### 3.2. Effect of ‘Remote’ Impedance Z_B_

**Figure 4:**
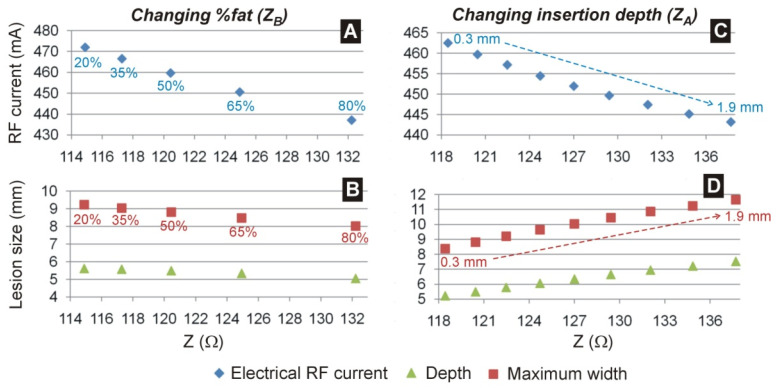
Electrical RF current (at the middle of the RF pulse, 15 s) (**A**,**C**) and lesion size (**B**,**D**) for different values of baseline impedance. While in panels (**A**,**B**) the baseline impedance varied due to the ‘remote’ impedance, specifically the percentage of fat around the organs (from 20 to 80%), in panels (**C**,**D**) the baseline impedance varied due to the ‘local’ impedance, specifically the electrode insertion depth in the myocardium (from 0.3 to 1.9 mm).

The increase in the percentage of fat relative to skeletal muscle was associated with an increase in baseline impedance. For instance, for a 0.5 mm insertion depth, baseline impedances ranged from 115 to 132 Ω when %fat varied from 20 to 80%. This trend was similar for any insertion depth, even for case of the electrode floating in the midpoint of the modeled cardiac chamber, with impedances ranging from 98 to 111 Ω. This increase in baseline impedance had an inverse effect on the RF current, which decreased from 472 to 347 mA (Figure 4A), which in turn implied slightly smaller lesion size: from 5.6 to 5.1 mm in depth, and from 9.2 to 8.0 mm in maximum width (Figure 4B). Both current and lesion size showed a linear relationship with baseline impedance (R^2^ > 0.98, *p* < 0.01), with rates of −2 mA/Ω for current, −0.03 mm/Ω for lesion depth and −0.07 mm/Ω for lesion maximum width.

### 3.3. Effect of ‘Local’ Impedance Z_A_

The increase in the insertion depth of the electrode in the cardiac wall was associated with an increase in basal impedance. For instance, for a 50% of fat–muscle mixture, baseline impedances ranged from 118 to 138 Ω when insertion depth varied from 0.3 to 1.9 mm. This trend was similar for fat–muscle mixture. This increase in baseline impedance also had an inverse effect on the RF current, which decreased from 463 to 443 mA (Figure 4C) but created progressively larger lesions: from 5.2 up to 7.5 mm in depth, and from 8.4 up to 11.6 mm in maximum width (Figure 4D). Both current and lesion size showed a linear relationship with baseline impedance (R^2^ > 0.98, *p* < 0.01), with rates of −1 mA/Ω for current, +0.12 mm/Ω for lesion depth and +0.17 mm/Ω for lesion maximum width.

### 3.4. Relationship between Current, Baseline Impedance and Lesion Size

**Figure 5:**
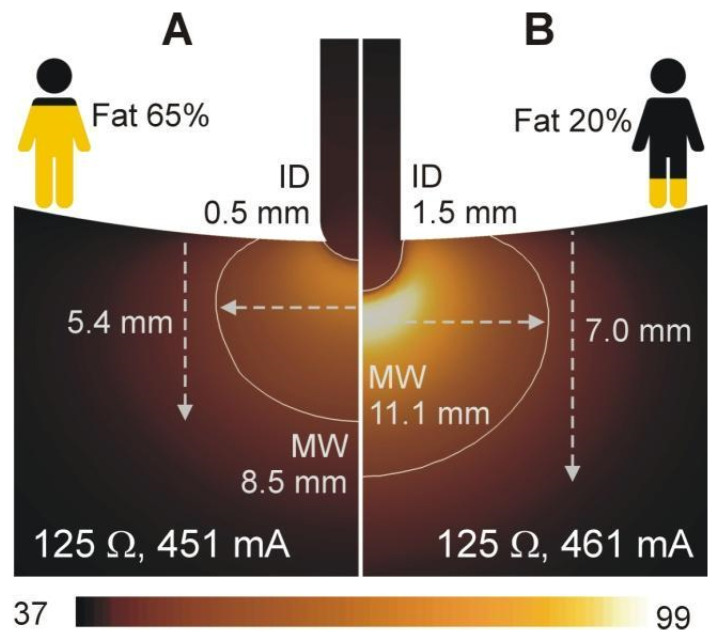
Temperature distributions (scale in °C) for two cases showing the same baseline impedance (125 Ω) despite different insertion depths of the electrode (0.5 mm in (**A**) vs. 1.5 mm in (**B**)) and fat percentages in the torso (65% in (**A**) vs. 20% in (**B**)). Note that the resultant lesion sizes are different despite identical baseline impedances. The RF current at the middle of the RF pulse (15 s) is 10 mA higher in the case of the largest lesion.

The simulation results showed that markedly different lesion sizes can be obtained for the same baseline impedance depending on the contribution of *Z_A_* and *Z_B_* to that baseline impedance. With a baseline impedance of ~120 Ω, the lesion depth varied from 6.2 mm (in the case of 20% fat and 0.9 mm insertion depth) to 5.5 mm (in the case of 50% fat and 0.5 mm insertion depth), despite a similar current (467 vs. 460 mA, respectively). Disparate lesion size results were observed with other simulations possessing similar baseline impedance values: while a lesion only 5.4 mm deep will be created in the case of 80% fat and 0.7 mm insertion depth, it could reach up to 7.6 mm deep in case of 35% fat and 1.9 mm insertion depth, all occurring with the same baseline impedance of ~135 Ω and similar current values (435 vs. 451 mA). Figure 5 shows the temperature distributions at the end of the RF pulse for two other cases sharing the same baseline impedance (125 Ω) but resulting in completely different lesion sizes due to the different contribution of *Z_A_* (related to the depth of insertion) and *Z_B_* (related to the amount of fat). The current at the middle of the RF pulse (15 s) is 10 mA higher in the case of the largest lesion (Figure 5B).

**Figure 6:**
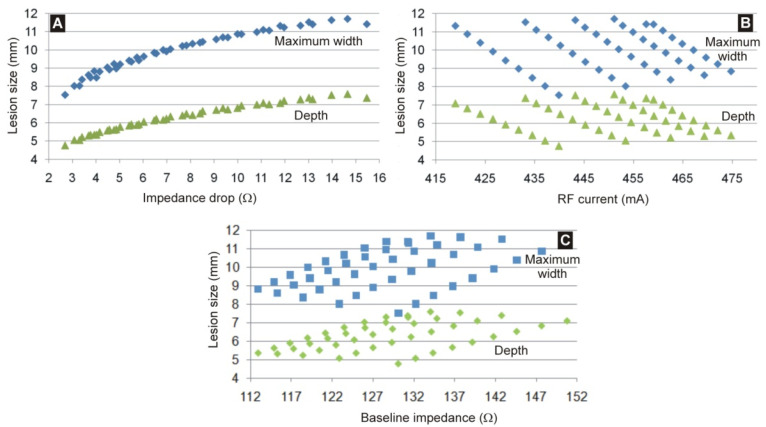
Relationship between lesion size (depth and maximum width) and impedance drop (**A**), total current delivered at 15 s (**B**) and baseline impedance (**C**).

Figure 6 shows the relationship between lesion size, impedance drop (the magnitude of reduction in impedance from baseline during RF delivery) and total current delivered at 15 s. Figure 6A shows a close linear relationship between lesion size and impedance drop: R^2^ > 0.96 for depth and R^2^ > 0.93 for maximum width (*p* < 0.01). The fit is even better in the case of a logarithmic regression, R^2^ > 0.99). In contrast, Figure 6B shows that there is a poor correlation between the total current and the lesion size (R^2^ < 0.1, *p* < 0.05), with markedly different possible lesions sizes for a given RF current. Figure 6C shows that there is also a poor correlation between the baseline impedance and the lesion size: R^2^ = 0.22 for maximum width and R^2^ = 0.25 for depth (*p* < 0.01).

### 3.5. Impact of Correcting Power According Baseline Impedance

**Figure 7:**
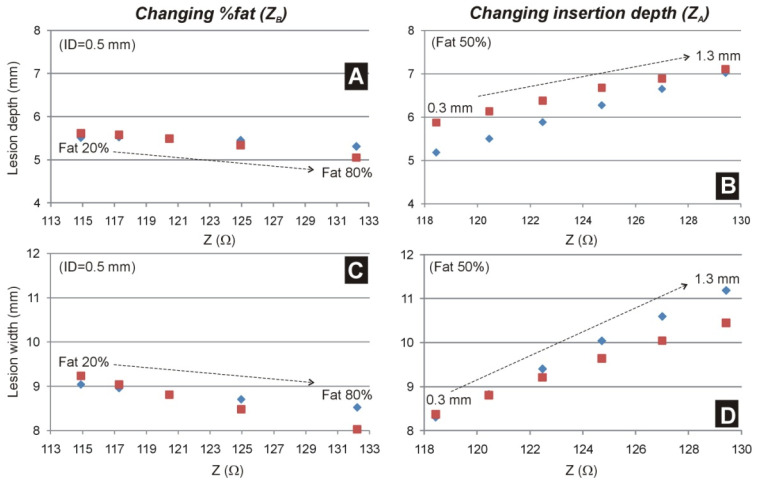
Lesion depth (**A**,**B**) and maximum width (**C**,**D**) computed for different baseline impedances with (blue marks) and without (red marks) ‘corrected’ power. While in (**A**,**C**) the scatter in baseline impedance is due to the percentage of fat in tissue rounded the heart ranging from 20 to 80% (i.e., changing *Z_B_*), in (**B**,**D**) the scatter in baseline impedance is due to the insertion depth of the electrode ranging from 0.3 to 1.3 mm (i.e., changing *Z_A_*).

Figure 7 shows the lesion size (depth and maximum width) computed for a range of baseline impedances in cases using 25 W or the corrected power using the formula 25 W × (*Z*/120 Ω). When varying *Z_A_* due to different electrode insertion depths, correcting the power does not create similar lesion sizes (see Figure 7B,D). Lesion sizes become less deep for low impedance values and wider for higher impedance values. In contrast, when the variations in baseline impedance are due to different percentages of fat in the tissues adjacent to the heart (affecting *Z_B_*, see Figure 7A,C), the power correction resulted in lesions more similar for different baseline impedances than in the case of no power correction.

## 4. Discussion

### 4.1. Main Findings

In this study, an in silico model using patient-derived anatomic measurements was used to test the hypothesis that baseline impedance predicts lesion size. The advantage of computer modeling is that both the local (*Z_A_*) and remote (*Z_B_*) components of the overall baseline impedance can be independently controlled to determine their relative effects on lesion formation. This model was then used to assess how differences in local versus remote impedance can affect lesion size for given impedance drops or system current during RF, as well as the performance of ‘corrected power’ to normalize lesion sizes for different baseline impedances. The principal findings of the study are:

(1) Increasing *Z_B_* by increasing the percent tissue fat resulted in higher baseline impedance, lower RF current, and smaller lesion formation controlling for the catheter insertion depth. In contrast, increasing *Z_A_* by increasing the insertion depth of the ablation electrode also resulted in higher baseline impedances and lower RF current but larger lesion sizes, controlling for the tissue fat percentage.
(2) Identical baseline impedances as result of different relative contributions of *Z_A_* and *Z_B_* can lead to similar observed RF currents but very different lesion sizes.
(3) Impedance drop during RF delivery showed a monotonic relationship with lesion size, while observed RF current during RF delivery did not show a predictable relationship with lesion size.
(4) When using ‘corrected power’ to account for baseline impedances, the correction formula only results in similar lesion sizes if differences in *Z* are due to variation in *Z_B_* rather than *Z_A_*.

This study has multiple clinical implications for RFCA. For a given patient, during an ablation procedure the dispersive patch is typically fixed into position and not moved, so for a given catheter position in the heart, *Z_B_* does not change, and any change in *Z* is expected to be due to *Z_A_*. As a result, most of the observed impedance drop during RF delivery is from a reduction in local impedance from local tissue heating, i.e., changes of **Z*_A_*. Operators can monitor indices that correlate to lesion size during RFCA, including impedance drop, RF current, force-time-integral, lesion size index (LSI), and the ablation index. Of these, the impedance drop, RF current, and LSI each depend on baseline impedance and/or current. However, the results here show that nearly identical baseline impedances or observed currents during RF delivery can result in different lesion sizes based on the relative contributions of local and remote impedances which could in turn provide variable results in using those indices to estimate adequate lesion formation. This is reflected in the poor correlation (R^2^ < 0.25) between the lesion size and both current and baseline impedance (see Figure 6) when considering all possible values of *Z_A_* and *Z_B_*. However, a good correlation is found (R^2^ > 0.98) if the relative contributions of the local and remote impedance are known in advance (see Figure 4), suggesting that pre-ablation measures aimed at estimating each of the two contributions might help predict the lesion size.

Barkagan et al. demonstrated in ex vivo swine heart RFCA that there was a negative correlation between baseline impedance and current squared, and lower baseline impedance was associated with larger lesions [1]. That study also demonstrated a better correlation between baseline impedance and current output than impedance drop with current output. That study, however, modulated the system impedance outside the ablation electrode-tissue interface (*Z_B_*) and did not account for local changes in local impedance (*Z_A_*), instead keeping the catheter contract force and orientation constant. In contrast, the study presented here showed a better association between lesion size and impedance drop (reflecting mostly local impedance drop) than with RF current since both local and remote impedances were accounted for. This demonstrates a limitation in using baseline impedance or RF current monitoring alone to guide ablation delivery. If a high baseline impedance is noted and power is therefore titrated up to achieve a higher overall RF current output, there may be a risk of overheating and steam pop if the baseline impedance is high due to high *Z_B_* but with an otherwise low *Z_A_*.

Both patient factors and ablation catheter factors must be considered when predicting lesion sizes based on impedance and current. Factors that affect *Z_A_* at the ablation electrode-myocardium interface include insertion depth (proportional to contact force) and catheter orientation relative to the myocardium. In addition to body fat percentage, *Z_B_* is also affected by the distance between the ablation site and dispersive pads, the properties of the other intervening tissues, and the size of the dispersive pad. Shapira-Daniels et al. showed that adding or repositioning the dispersive patch in order to change the system impedance (by modulating *Z_B_*) can be used to increase RF current output to produce greater impedance drops and successful ablations in patients with deep intramural substrate [3]. In agreement with that study, the results presented here show that reductions in remote impedance can lead to larger lesions for a given catheter position and power delivered.

There are now commercially available ablation catheters that can estimate the ‘local’ impedance at the catheter-myocardial interface by using microelectrodes near the tip of the catheter to detect electrical potential changes in a small field created by a nonstimulatory current between the tip and ring electrodes and solving for impedance using Ohm’s Law [16]. As expected, when assessed with ultrasound imaging, increased myocardial contact led to increased local impedance compared to the catheter tip floating in the blood pool, which is in concordance with our model. In a swine model, larger local impedances drop during RFCA led to large lesion volumes with a closer correlation between local impedance drop and lesions size than overall system impedance drop with lesion size [17]. In a study of 25 patients undergoing RFCA for atrial fibrillation, larger local impedance drops were observed compared to overall system impedance drops and baseline local impedance was correlated with the local impedance drop during RF application. That result supports the idea that local tissue heating leading to lower *Z_A_* drives the overall impedance drop, and higher baseline local impedance implies better tissue contact and more tissue heating resulting in larger lesions [18].

### 4.2. Limitations

This study has limitations inherent to any in silico model. Computer simulations make numerous assumptions in order to allow for computations to be feasible, and models cannot account for all physical factors. This study used measurements from a small number of patients at a single plane in the chest to form the anatomic schema, and this study only modeled ablation at the posterior LA (an important site of ablation during atrial fibrillation ablation), limiting the generalizability to other types of RFCA in other chambers. For simplicity the model only assessed changes in remote impedance due to tissue fat percentage and local impedance due to catheter insertion depth. Despite that, the results can be well-explained in physical terms and help to explain the relationship between electrical impedance and lesion size during RFCA. Moreover, the findings about how the two components of the baseline impedance (local and remote) can contribute to the lesion size can be extended by operators to make other modifications to the electrical circuit to improve lesion formation during RFCA, including decreasing ‘remote’ impedance by adding more dispersive patches (which enlarges the cross-section of the electrical circuit) or repositioning the patch to a point closer to the heart (which shortens the length of the impedance). Note that although the concept of local and remote impedance has been widely used by researchers in the form of distributed element electrical circuits to explain the physics of RFCA [1,2,3], this is the first study in which these concepts are expanded to the case of a continuous medium based on medical imaging, which better represents the real situation.

## 5. Conclusions

Effective lesion delivery during RFCA requires understanding the disparate impacts of both local and remote impedances to the overall power delivery to the myocardium. An in silico model of RFCA demonstrates that otherwise identical baseline impedances and observed RF currents can lead to markedly different lesion sizes with different relative contributions of local and remote impedances to the electrical circuit. These findings emphasize the importance of multiparameter, real-time monitoring during RFCA, as no single metric or index is sufficient to ensure efficient and safe RF energy delivery.

## Figures and Tables

**Table 1:** Thermal and electrical characteristics of the elements employed in the model *.

Element/Material	*σ* (S/m)	*k* (W/m·K)	*ρ* (kg/m^3^)	*c* (J/kg·K)
Electrode/Pt-Ir	4.6 × 10^6^	71	21,500	132
Catheter/Polyurethane	10^−5^	23	1440	1050
Cardiac wall/Myocardium	0.281	0.56	1081	3686
Cardiac chamber/Blood	0.748	Thermal problem not solved		
Muscle	0.446	0.49	1090	3421
Subcutaneous fat (infiltrated fat)	0.0438	0.21	911	2348
Lungs	0.215	0.39	722	3886
Spine, sternum/bone *	0.055	0.315	1543	1793

* σ, electrical conductivity (at 500 kHz); k, thermal conductivity; ***ρ***, density; and c, specific heat (all assessed at 37 °C in case of tissue and blood). Electrical conductivity of tissue was assumed to be increase by +1.5/°C until 100 °C, and then drastically decreased two orders of magnitude between 100 and 105 °C to mimic the desiccation associated with vaporization. Heat latent associated with change phase (from liquid to gas) was also included by making the ***ρ*** × c 400 larger between 99 and 100 °C.

**Table 2:** Morphological data from the CT scans and percentage of fat relative to mixture skeletal muscle-fat surrounding the organs.

Pt #	Sex	Age (years)	BMI (kg/m^2^)	BSA (m^2^)	Sternum-LA (mm)	LA-Spine (mm)	%Fat
1	M	33	34.18	2.41	163	114	52
2	M	58	39.38	2.27	159	139	65
3	F	38	37.73	2.37	170	110	72
4	M	84	29.52	1.87	142	105	78
5	F	43	30.30	1.68	149	89	63
6	M	61	26.41	1.94	140	109	61
7	F	90	25.06	1.73	140	114	78
8	M	55	36.24	2.58	171	125	73
9	M	67	29.40	2.14	161	160	62
10	M	72	34.96	2.49	161	128	78
11	M	66	29.98	2.02	174	129	65
12	F	61	24.80	1.82	137	112	51
13	M	73	26.66	2.17	165	106	54
14	F	49	35.48	1.96	146	113	81
15	M	75	22.96	2.01	149	100	55
16	M	86	31.48	2.05	146	117	59
17	M	59	30.78	2.24	171	134	36
18	M	79	30.72	2.09	168	127	40
19	M	69	37.31	2.50	172	155	63
20	F	63	17.38	1.37	110	73	29
Mean		64.1	30.54	2.09	154.7	118.0	61
SD		15.5	5.59	0.31	16.2	20.4	14
Min		90	39.38	2.58	174	160	81
Max		33	17.38	1.37	110	73	29

Sternum-LA: Distance between sternum and posterior left atrium wall. LA-Spine: Distance between spine and posterior left atrium wall.

## Data Availability

Not applicable.
